# Crosstalk Between Long Non-coding RNAs, Micro-RNAs and mRNAs: Deciphering Molecular Mechanisms of Master Regulators in Cancer

**DOI:** 10.3389/fonc.2019.00669

**Published:** 2019-07-25

**Authors:** Eduardo López-Urrutia, Lilia P. Bustamante Montes, Diego Ladrón de Guevara Cervantes, Carlos Pérez-Plasencia, Alma D. Campos-Parra

**Affiliations:** ^1^Unidad de Biomedicina, FES-IZTACALA, Universidad Nacional Autónoma de México, Tlalnepantla de Baz, Mexico; ^2^Decanato, Ciencias de la Salud, Universidad Autónoma de Guadalajara, Zapopan, Mexico; ^3^Laboratorio de Genómica, Instituto Nacional de Cancerología (INCan), Mexico City, Mexico

**Keywords:** cancer, long non-conding RNAs, miRNAs, mRNAs, angiogenesis, vascularization, vasculogenic mimecry

## Abstract

Cancer is a complex disease, and its study requires deep understanding of several biological processes and their regulation. It is an accepted fact that non-coding RNAs are vital components of the regulation and cross-talk among cancer-related signaling pathways that favor tumor aggressiveness and metastasis, such as neovascularization, angiogenesis, and vasculogenic mimicry. Both long non-coding RNAs (lncRNAs) and micro-RNAs (miRNAs) have been described as master regulators of cancer on their own; yet there is accumulating evidence that, besides regulating mRNA expression through independent mechanisms, these classes of non-coding RNAs interact with each other directly, fine-tuning the effects of their regulation. While still relatively scant, research on the lncRNA-miRNA-mRNA axis regulation is growing at a fast rate, it is only in the last 5 years, that lncRNA-miRNA interactions have been identified in tumor-related vascular processes. In this review, we summarize the current progress of research on the cross-talk between lncRNAs and miRNAs in the regulation of neovascularization, angiogenesis and vasculogenic mimicry.

## Introduction

Cancer is a serious worldwide health problem that affects the health of all human cultures. Prostate and breast cancer rank as top prevalent cancer types in men and women, respectively (1). Cancer has been defined as a complex, heterogeneous, and multifactorial disease that occurs by the presence of driver mutations that leads to the activation of proto-oncogenes and corresponding inactivation of tumor suppressors. This provokes a switch in cell functions that ultimately leads to the hallmarks of cancer (2).

In addition to mutations in protein-coding genes, recent advances in molecular oncology have described the aberrant expression of non-conding RNAs such as micro-RNAs (miRNAs) and long-non-coding RNAs (lncRNAs) (3, 4). Both molecules are well-established as master regulators of multiple protein-coding genes (5). Among other functions, lncRNAs can act as molecular decoys, sequestering miRNAs, and consequently, inhibiting their interaction with their target messenger RNAs (mRNA) (6, 7). This way, lncRNAs regulate a wide range of biological processes through their crosstalk with miRNAs that, in turn, regulate mRNAs (8). Since these crosstalking molecules are so closely related, abnormal expression of lncRNAs interferes with mRNA expression patterns creating a dysregulation that can culminate in cancer development (9). In the present review, we summarize the recent studies on the lncRNA-miRNA-mRNA crosstalk in order to provide insight into the complexity of the molecular mechanism that underlies neovascularization, angiogenesis, and vasculogenic mimicry.

## MicroRNAs

Micro-RNAs (miRNAs), are small single-stranded 18–25 nucleotide RNAs. They play key roles in biological processes such as development, stem cell differentiation, and tissue identity through negative regulation of mRNA transcripts (10). Twenty-six years after their discovery, the number of studies that describe their role in cancer is still increasing, so they have earned their place as diagnostic, prognostic, and therapeutic biomarkers (5).

The earliest report on miRNAs was made by the Ambros lab (11). Lin-4 is a 22-nucleotide RNA with sequence complementarity to a region of the 3′UTR in the lin-14 mRNA, which inhibits lin-14 mRNA from being translated. However, it was not until 2001 that Ambros coined the term miRNA when describing a number of small RNAs with a role in gene regulation that had been recently identified in *C. elegans* (12).

Most miRNAs are transcribed in the form of a primary miRNA (pri-miRNA) by RNA polymerase II (Pol II), then processed by the nuclear microprocessor (comprised by the Ribonuclease II DROSHA, and DGCR8) to form the pre-miRNA, which is later exported to the cytoplasm by means of an Exportin-5-Ran-GTP-shuttle protein. In the cytoplasm, DICER binds to the pre-miRNA and cleaves it to its final 22 nt mature form that associates with AGO 2 to form the RNA-induced silencing complex (RISC). MiRNAs function through sequence complementary: within the RISC, the miRNA binds the target mRNA 3′UTR and, based on the degree of complementarity, leads to full mRNA degradation or blocking of the ribosomal machinery, both result in gene silencing (13).

The first reported miRNAs contributing to cancer were miR-15/16 in Chronic Lymphocytic Leukemia (CLL). Under normal conditions, both miRNAs repress antiapoptotic Bcl-2 protein, which is overexpressed in CLL (14). Since then, several miRNAs associated with cancer have been described. Ongoing research on miRNAs and their role in cancer development shows their great potential as biomarkers, therapeutically targets or even as potential therapies, restoring function of tumor suppressor miRNAs (10).

## Long-non-coding RNAs

Transcripts that do not encode proteins and are more than 200 nucleotides in length, are termed long non-coding RNAs (lncRNA) (15). Many of them resemble mRNAs in aspects such as being 5′capped, spliced, and polyadenylated; but differ in a shorter overall length, fewer but longer exons, and lower expression levels (16).

Transcription of lncRNAs is similar to other eukaryotic RNAs, transcribed by RNA Pol II from bidirectional promoters (15). These promoters are often enriched in H3K27ac, H3K4me3, and H3K9ac modified histones and are repressed by remodeling complexes such as Swr1, lsw2, Rsc, and Ino80; therefore, SWI/SNF complex activity is needed to promote transcription initiation. After being transcribed, their structure is unstable, and they are subject to nuclear exosome or cytosolic non-sense-mediated decay, so their half-life is short (<2 h) compared to miRNA (48-h half-life). It is still unknown whether this mechanism is followed by all lncRNAs (17).

LncRNA classification relies on the empirical attributes originally used to detect them such as size, localization, and function (18) although it is yet to reach a universally recognized consensus. The latest classification by the genomic consortium GENCODE categorizes them according to their genomic location in five groups: (1) Antisense RNAs: encompasses RNAs that are transcribed from the antisense strand near an exon of a protein-coding locus; (2) Long intergenic non-coding (LincRNA): includes RNAs that are transcribed from intergenic loci; (3) Sense overlapping transcripts: transcripts that comprehend a coding gene inside an intron on the same strand, (4) Sense intronic transcripts: comprises transcripts that are encoded in introns of coding genes, (5) Processed transcripts: RNAs that do not contain an ORF and cannot be otherwise classified (19).

Due to their ability to interact with DNA, RNA, and proteins, lncRNAs are able to regulate very diverse cellular processes such as chromatin modification, transcription, post-transcriptional modifications, scaffolding, and post-transcriptional mRNA regulation. Consequently, lncRNAs can be found in equally diverse subcellular locations: nucleus, subnuclear domains, and cytoplasm (6, 7).

The existence of lncRNAs was first reported in the early 1990s with the discovery of H19 and Xist in mouse (20, 21). Subsequently, novel lncRNAs candidates were identified and their true relevance in human biology and disease was revealed (22, 23). A role in cancer for lncRNAs was only suggested last decade, when HOTAIR (24) and H19 (25) were found to modify the transcriptional landscape through chromatin modification. Since then, many reports have concurrently established a role for lncRNAs in cancer development (26). Moreover, they are uniquely promising cancer biomarkers since they are easily detectable in body fluids (27, 28).

## LncRNA-miRNA Interaction

Besides the regulation that both miRNAs and lncRNAs alone exert on mRNAs, it has been reported that they interact with one another, further modulating their influence in the transcriptome. These interactions lead to miRNA-triggered RNA decay, competition between miRNAs and lncRNAs for the same mRNA target, miRNA generation from lncRNAs, and lncRNAs acting as decoys for miRNAs [extensively reviewed in (29)].

**Figure 1:**
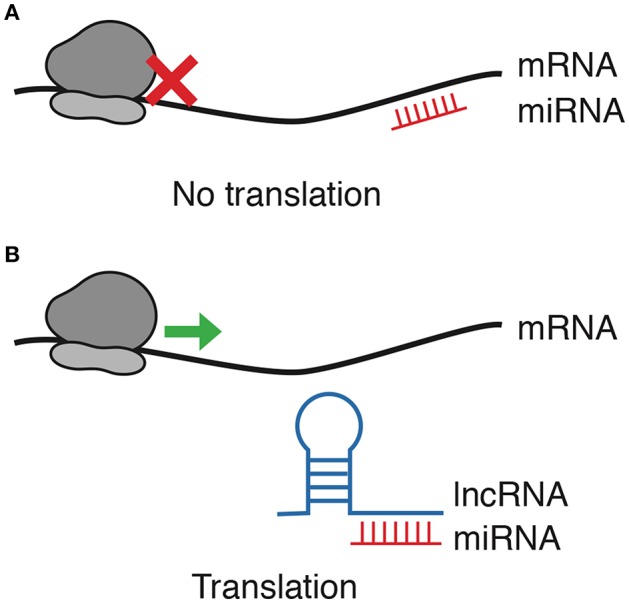
lncRNA/miRNA/mRNA axis regulation. Among other interaction forms between ncRNAs and mRNAs, this review focuses only on lncRNAs blocking the negative regulation exerted by miRNAs. **(A)** miRNAs block translation by binding mRNA. **(B)** As lncRNAs function as decoys for miRNAs, mRNA translation is allowed.

Multiple reports show that the latter is the most prevalent lncRNA-miRNA interaction in cancer. LncRNAs that bind miRNAs and prevent their interaction with their target are regarded to as competitive endogenous RNAs (ceRNAs), decoys or sponges (30); since they prevent miRNAs from completing their regulatory function, lncRNAs acting as sponges are, effectively, positive regulators of mRNA transcripts (Figure 1). Interestingly, most lncRNAs capture miRNAs using regions close to their 3′ end named miRNA Response Elements (MRE), which are complementary with the Ago binding sites present in most miRNAs (31). It is relevant to mention that, while most RNA-RNA interaction reports come from strictly controlled experiments, the exact relationship between the plethora of RNAs in the cell—and thus the efficiency of competitive endogenous interactions—remains to be entirely understood in pathological models, which often present strong dysregulation of specific competing endogenous RNAs (32).

Prediction of these mechanisms has gained importance in the latest years due to the broad impact of the lncRNA-miRNA regulation. This has led to the development of bioinformatic tools such as MechRNA (33), RNAHybrid (34), RNADuplex (35), and RNAcofold (36) among others, that aim to elucidate lncRNA-miRNA interactions. Likewise, searchable repositories of lncRNA-miRNA interactions such as miRcode (37) are working to facilitate the study of RNA regulation through information. At the time of writing, experimental validation of lncRNA-miRNA interactions is necessary (38).

The role of lncRNAs is certainly complex. For instance, it was recently reported that UCA1 binds the 3′UTR of mRNAs to prevent their degradation by miRNAs, constituting a RNA-based regulatory signaling, which regulates cancer-linked pathways (39).

In the following sections, we review experimentally validated lncRNA-miRNA interactions with a role in tumor development processes. The path toward a full understanding of the ncRNA regulation networks is still long, but we are convinced that this is an exciting time to study regulatory RNAs.

## Angiogenesis

Angiogenesis is the process that generates capillary networks from pre-existing blood vessels in response to the need of nutrients in a given tissue region (40). It occurs throughout development and adult life, precisely controlled by a network of angiogenesis activators such as VEGF and inhibitors such as VASH2 (41). Tumor cells demand nutrients and thus modulate angiogenesis to their advantage altering the delicate activator-inhibitor balance (42). In the reviewed literature, we found that the VEGF-A mRNA participates in at least four lncRNA/miRNA/mRNA axes, albeit in different cancers. The TUG1/miR-299/VEGF-A axis increased angiogenesis in glioblastoma (43); LINC00668/miR-297/VEGF-A axis led to increased cell proliferation in oral squamous cell (44); and AK131850/miR-93-5p/VEGF-A promoted differentiation, migration and tube formation of endothelial progenitor cells (45).

Interestingly, miR-199a regulates both VEGF-A and its activating transcription factor, HIF-1a; thus, both of them are upregulated by Snhg1 lncRNA when it blocked miR-199a in a dual action Snhg1/miR-199a/VEGF-A&HIF-1a axis in bone marrow microvascular endothelial cells, promoting their proliferation (46). A somewhat similar mechanism was observed in HUVEC cells, where MALAT1 lncRNA antagonized miR-320a and upregulated the transcription factor FOXM1 (47), which also activates VEGF-A transcription. More studies are still needed to confirm whether VEGF upregulation is a common mechanism, attained by different lncRNAs in different tumors or this regulation has a high degree of redundancy and each of the investigated lncRNAs are active in other tumors as well.

Some other angiogenesis-related signaling proteins are upregulated by lncRNAs as well. For instance, VASH2 has been shown promote angiogenesis in tumors (48) and H19 lncRNA—highly expressed in glioma cells—upregulates VASH2 through the H19/miR-29a/ VASH2 axis. Zheng et al. (49) found that ANGPT2, a pro-angiogenesis signaling molecule is targeted by miR-26b, and upregulated in HUVEC cells by the sponge activity of PVT1 over miR-26b (50). This same PVT1-miR-26b interaction results in the upregulation of CTGF, a pro-inflammatory mediator with a role in promoting angiogenesis (51).

Interestingly, lncRNA-driven upregulation of angiogenesis has been observed in at least one non-tumoral context. The WTAPP/miR-3120-5p/MMP-1 axis, promotes angiogenesis in endothelial progenitor cells (52). Since, MMP-1 has an established role in cancer development (53), it is likely that WTAPP1 also promotes angiogenesis in tumors.

## Neovascularization

Neovascularization is a mechanism through which new blood vessels are made from preexistent ones, this process is coordinated by angiogenesis inductors and inhibitors following endothelial cell proliferation and migration (54). Developing tumors obtain the required nutrients and oxygen from neighboring blood capillaries; nonetheless, since the diffusion distance of oxygen is 100–200 μm, the generation of new blood vessels is necessary for tumors larger than 1–2 mm (55). In this hypoxic environment, the HIF-1 induces the expression several growth factors (e.g., HGF) and VEGF to promote hypervascularization (56). An important distinction between neovascularization and angiogenesis is that the latter is a requirement for tumor progression accelerating the tumor development (57). In the reviewed literature, we found only 5 papers published from 2015 to 2017, describing the lncRNA-miRNA-mRNA crosstalk orchestrating this mechanism.

Deng et al. determined the role of the CCAT1/Let-7/c-myc axis in hepatocellular carcinoma. High expression of CCAT1 was associated with larger tumor size, microvascular invasion and alpha fetoprotein (58). Both HMGA2 and c-myc are let-7 targets; however, only c-myc was observed up-regulated while CCAT1 was stably overexpressed in SMMC-7721 cells. Deng et al. concluded that CCAT1 regulates let-7 and this, in turn regulates c-myc in order to coordinate proliferation and migration events in hepatocarcinoma (58).

Dong et al. through *in vitro* and *in vivo* analysis demonstrated the participation of TUG1/miR-34a-5p/VEGF-A axis in hypervascularity and hepatoblastoma progression (59). In a xenograft model, TUG1 knockdown lead to a significant tumor reduction up to 28% compared to the control group. Significantly diminished VEGF-A levels indicated that miR-34a-5p is a miRNA target of TUG1. At the same time, VEGF-A was a mRNA target of miR-34a-5p (59). Thus, the TUG1/miR-34a-5p/VEGF-A axis contributes to unusual hypervascularity in hepatoblastoma.

Glioma is a well-studied model for neovascularization (60). Significant H19 overexpression in microvessels from glioma specimens vs. normal brain microvessels leads to enhanced proliferation, migration, and tube formation with major tubule length and number of branches in H19 overexpressed glioma-associated endothelial cells. Besides, H19 overexpression decreased the miR-29a level and promoted the VASH2 overexpression. H19 acts a sponge for miR-29a; moreover, H19 knockdown promoted miR-29a overexpression and decreased VASH2 protein level in consequence diminished proliferation, migration and tube formation, establishing the H19/miR-29a/VASH2 axis (60). In another report from glioma cells, cell growth was arrested by H19 expression inhibition. MiR-140 was detected as a H19 miRNA-target, as suggested when H19 overexpression and miR-140 downregulation were determined and was corroborated by luciferase assay. Simultaneously, it was determined that iASPP—previously reported to promote cancer cell growth—was a direct target of miR-140 (61). Also, it was reported that PVTI lncRNA and miR-186 expression were inversely correlated in glioma. Functional analyses showed that PVTI stable transfection of glioma vascular cells lines favored proliferation, migration and tube formation. Likewise, miR-186 knockdown supported proliferation, migration and tube formation of glioma vascular endothelial cells; miR-186 inhibits expression of ATG7 and Beclin I, essential proteins to autophagy-lysosome formation. The authors suggested that PVTI and miR-186 could be deliver new objectives for glioma anti-angiogenic therapy (62). Together, these reports strongly suggest and important role for H19 in neovascularization in glioma through at least three lncRNA/miRNA/mRNA axes.

## Vasculogenic Mimicry

Vasculogenic mimicry (VM) was first described by Maniotis et al. They defined it a vascular-like structure which can mimic the embryonic vascular network (microcirculatory channels comprised of extracellular matrix) to sustain tumor tissue providing it with plasma and red blood cells (63). An important distinguishing characteristic is that vasculogenic mimicry resembles the embryonic vasculogenesis processes, suggesting that tumor cells can be converted back to an undifferentiated, embryonic-like phenotype to provide nutrients that ensure tumor growth in hypoxic environment (64). This mechanism has been observed in several tumors such as melanoma, ovarian, breast, prostate, osteosarcoma, bladder, colorectal, and lung cancers, where it plays an important role in invasion and metastasis; thus, patients with VM have a worse prognosis (65).

Several key molecules have been reported associated with this process including Notch1, MMP-2, MMp-9, vimentin (66), VE-cadherin, EphA2, FAK, PI3-Kinase (67), VEGF, endostatin, TGF-ß1 (68), Dickkopf-1 (69), maspin (70), laminin, CD44, thrombospondin 1, and cyclin E2 (64), among others. The participation of master regulators such as miRNAs and lncRNAs has not gone unnoticed, although our literature review yielded only five papers on the miRNAs/lncRNAs/mRNAs cross-talk and VM regulation.

Gao et al. observed HOXA-AS2 overexpression in glioma cell lines and tissues. HOXA-AS2 knockdown lead to underexpression of MMP-9, MMP-2 and VE-cadherin proteins and, consequently to VM inhibition; HOX-AS2 turned out to sponge miR-373, which, interestingly, did not target MMP-9, MMP-2, or VE-cadherin but EGFR. Furthermore, HOXA-AS2 knockdown favored miR-373 expression and EGFR downregulation in U87 and U251 cell lines. Xenograft and orthotopic models further demonstrated that HOXA-AS2 knockdown plus pre-miR-373 produced the smallest tumors, the longest survival time and the lowest VW densities (71).

We found that TWIST1 has an important role in VM, as it participates in at least two lncRNA-miRNA axes. In glioma, in is upregulated by LncRNA LINC00339 via miR-539-5p. Functional analysis revealed that overexpression of miR-539-5p inhibited the viability, migration, invasion and tube formation of the cell lines by downregulating TWIST1. Moreover, TWIST1 binds to the promoter of MMP-2 and MMP-14, both involved in VM formation. In xenograft models with knockdown LINC00339 and pre-miR-539-5p, smaller tumors and longer overall survival supported the LINC00339/miR-539-5p/TWIST1 axis (72). In triple-negative breast cancer (TNBC), the regulation of TWIST1 is through miR-430-3p which, in turn, is regulated by TP73-AS1. Both an inverse correlation between TP73-AS1 and miR-430-3p expression, and the interaction between miR-490-3p and TWIST1 were found in MDA-MB-231 cells. Interestingly, it was observed that the enforced expression of TWIST1 and the inhibition of miR-430-3p increased VM formation (73).

Zhao et al. reported that lncRNA n339260 overexpression was associated with the presence of metastasis, shorter overall survival and with MV in hepatocellular carcinoma (HCC) patients (74). LncRNA n339260 resulted critical to induce stem-like characteristics and VM formation; also, its expression was correlated with c-Myc, SOX2 and Nanog expression, which are pluripotency-maintaining molecules. Interestingly, the target miRNAs of n339260 were miR-31-3p, miR-30e-5p, miR-519c-5p, miR-520c-5p, miR-29b-1-5p, and miR-92a-1-5p, which were detected by microarray in HepG2 cells transfected with this lncRNA (74).

The MALAT1/miR-145-5p/NEDD9 axis was described in lung cancer: MALAT1 sponges miR-245-5p to amplify NEDD9 expression. Interestingly, MALAT1 is induced by the ERβ, a novel role for this receptor in lung cancer progression in female patients. NEDD9 also plays an important role in metastasis through TGFβ signaling pathway. This axis was analyzed in xenograft models and it was observed that ERβ promoted metastasis via MALAT1/miR-145-5p/NEDD9 signal (75).

## Conclusion and Perspectives

Angiogenesis, neovascularization and VM, as tumor progression and metastasis mechanisms, are becoming more important as sources of biomarkers and therapeutic targets, as the authors of several of the reviewed papers point out. On the other hand, the nuances of lncRNA/miRNA/mRNA regulation are not analyzed when ncRNA expression profiles are sought (76), and global analyses of this regulation mechanisms are still scarce [e.g., (77)]. So we considered it important to summarize current knowledge on the lncRNA/miRNA/mRNA axis regulation regarding angiogenesis, neovascularization, and VM, as it is still limited and deserves further scrutiny, perhaps due to the high methodological requirements. Upon analyzing the PubMed-listed papers, we found few studies that address lncRNA/miRNA/mRNA axis regulation of these nutrient supply processes.

**Figure 2:**
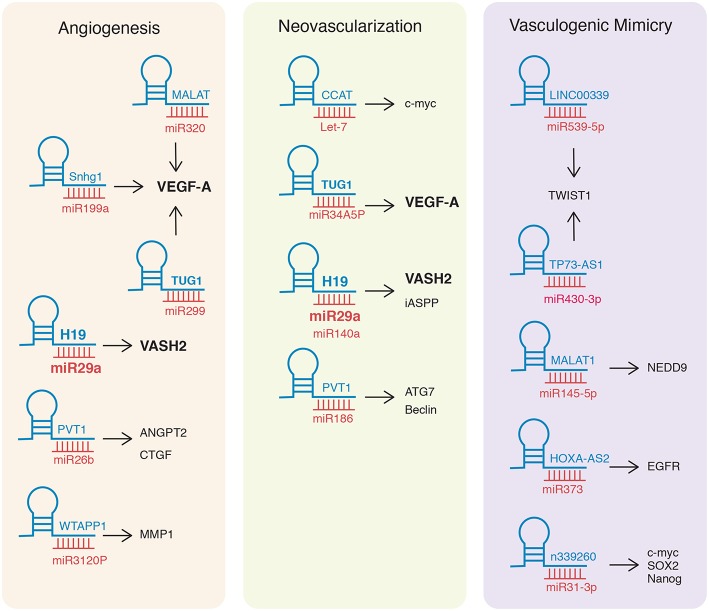
lncRNA/miRNA/mRNA axes involved in Angiogenesis, Neovascularization, and Vasculogenic Mimicry. The main lncRNA/miRNA/mRNA axes reported in the literature are depicted. lncRNAs (blue) bind miRNAs (red) and upregulate (arrows) mRNAs (black). Bold characters denote axes shared between processes.

So far, available information shows that lncRNA H19 is involved in angiogenesis and neovascularization, although in diverse manners. The sharing of the H19/miR-29a/VASH2 axis by both angiogenesis and neovascularization hints at a master regulation role for H19 and VASH2 (Figure 2). Interestingly, vasculogenic mimicry did not share any lncRNA/miRNA/mRNA axes with angiogenesis or neovascularization, which makes it reasonable to speculate that this is a specific molecular process and suggests pivotal role for it in aggressive tumors.

Our review has shown us the important role of lncRNA/miRNA/mRNA regulation in cancer development, an open area of opportunity that grants broader and deeper exploration in the following years.

## Author Contributions

AC-P, CP-P, and EL-U contributed to the conception of the article. AC-P, CP-P, EL-U, LB, and DL wrote and revised the final manuscript and agreed on its submission to this journal.

### Conflict of Interest Statement

The authors declare that the research was conducted in the absence of any commercial or financial relationships that could be construed as a potential conflict of interest.
